# Isolation of blue-green eggshell pigmentation-related genes from Putian duck through RNA-seq

**DOI:** 10.1186/s12864-019-5436-4

**Published:** 2019-01-19

**Authors:** Ding-Ping Bai, Xin-Yu Lin, Yan Wu, Shi-Ye Zhou, Zhong-bin Huang, Yi-Fan Huang, Ang Li, Xiao-Hong Huang

**Affiliations:** 1 Fujian Key Laboratory of Traditional Chinese Veterinary Medicine and Animal Health, Fuzhou, 350002 China; 2 Key Laboratory of Animal Embryo Engineering and Molecular Breeding of Hubei Province, Wuhan, 430064 China; 3 Shishi Conservation and Research Centre of Waterfowl Genetic Resources, Quanzhou, 362700 China; 4 University Key Laboratory for Integrated Chinese Traditional and Western Veterinary Medicine and Animal Healthcare in Fujian Province, Fuzhou, 350002 China

**Keywords:** Eggshell colouration, Putian duck, Transcriptome, Blue-green eggshell colour

## Abstract

**Background:**

The diversity of avian eggshell colour plays important biological roles in ensuring successful reproduction. Eggshell colour is also an important trait in poultry, but the mechanisms underlying it are poorly understood in ducks. This study aimed to provide insights into the mechanism of blue-green eggshell colour generation.

**Results:**

Here, white-shelled ducks (HBR) and blue-green-shelled ducks (HQR) were selected from Putian black ducks, and white-shelled ducks (BBR) were selected from Putian white ducks. Transcriptional changes in the shell gland were analysed using RNA-sequencing on the Illumina HiSeq 2500. Twenty-seven individual cDNA libraries were sequenced and generated an average of 7.35 million reads per library; 70.6% were mapped to the duck reference genome, yielding an average of 13,794 genes detected, which accounted for approximately 86.39% of all 15,967 annotated duck genes. A total of 899 differentially expressed genes (DEGs) were detected between the HQR and BBR groups, and 373 DEGs were detected between the HQR and HBR groups. We analysed the DEGs in the HQR-vs-BBR and HQR-vs-HBR comparisons. None of these DEGs were directly involved in the eggshell pigmentation process in HQR-vs-HBR, while UDP-glucuronosyltransferase 2A2 (UGT2A2) and UDP-glucuronosyltransferase 1–1-like (UGT1–1-like), which participate in biliverdin breakdown, were two of the DEGs in HQR-vs-BBR. In the RT-qPCR results, delta-aminolevulinic acid synthase 1 (ALAS1) and EPRS glutamyl-prolyl-tRNA synthetase were significantly upregulated in the HBR group compared with the HQR and BBR groups (*P* < 0.05). Haem oxygenase (HMOX1) was significantly downregulated in BBR compared with HQR and HBR (*P* < 0.05). Biliverdin reductase A (BLVRA), GUSB glucuronidase beta, cytochrome c-type haem lyase, protohaem IX farnesyltransferase and UGT2A2 were significantly upregulated in HBR and BBR compared with HQR (*P* < 0.05).

**Conclusions:**

We conducted a comparative transcriptome analysis of the shell glands of Putian white ducks and Putian black ducks. None of the differentially regulated pathways were directly involved in the eggshell pigmentation process in the HQR-vs-HBR comparison, while 2 DEGs related to biliverdin breakdown were found in HQR-vs-BBR. Based on the RT-qPCR results, we can speculate that both HQR and HBR can produce biliverdin, but HBR cannot accumulate it. Compared with HQR, BBR produced less biliverdin and did not accumulate it.

## Background

Bird eggshell colour displays enormous diversity and has multiple functions, such as avoiding predation [1], strengthening the structure of the shell [2], filtering harmful solar radiation [3] and decreasing trans-shell microbial contamination [4]. Colourful biological pigments are deposited in the shell gland during eggshell formation, resulting in different eggshell colours. Protoporphyrin IX, biliverdin and Zn-biliverdin chelate are three main pigments responsible for eggshell colouration. Protoporphyrin IX is an immediate precursor of haem, which causes reddish or brown background eggshell colour [5]. Biliverdin is a byproduct of the breakdown of haemoglobin and gives eggshells a blue or green colour [6]. Protoporphyrin is a pro-oxidant [7], while biliverdin possesses antioxidant activity [8]. The sexually selected eggshell colouration (SSEC) hypothesis assumes a positive association between female quality and the amount of biliverdin deposited into the eggshell during reproduction [9]. At the same time, darker and brown eggshells reflect poorer maternal condition [10, 11]. Identifying the molecular mechanisms involved in eggshell colouration is key to analysing the selection and evolution of this trait.

White and blue-green are the two major eggshell colour phenotypes in ducks, and eggs with a blue-green colour are more acceptable to consumers in Asia [12]. Improving egg colour in domestic poultry has long been of commercial interest, and the mechanism of eggshell colouration in laying hens has been studied previously. Most researchers have suggested that protoporphyrin IX and biliverdin are first synthesized in the shell gland and then secreted and deposited into the eggshell layers [13–15]. Some researchers believe that a specific enzymatic system is present in the shell glands of birds laying blue-green eggshell colour eggs that is absent from other birds [16]. The biosynthetic pathway of eggshell colour-related pigments is well established. Briefly, *SLC25A38* (solute carrier family 25, member 38) imports glycine into the mitochondrial matrix from the cytosol [17]. ALAS1 catalyses the rate-limiting step in the condensation of succinyl coenzyme and glycine to delta-aminolevulinic acid (ALA) [18]. Following its synthesis, ALA is exported to the cytosol and converted to coproporphyrinogen III [19]. *ABCB6* belongs to the ATP-binding cassette family that transports coproporphyrinogen III back to mitochondria [20]. Coproporphyrinogen oxidase (CPOX) then catalyses the conversion of coproporphyrinogen III to protoporphyrinogen IX [21], and protoporphyrinogen IX is oxidized into protoporphyrin IX via protoporphyrinogen oxidase [21], which gives eggshells a brown or pink colour [5]. Ferrochelatase (FECH) catalyses the terminal step of haem biosynthesis by inserting ferrous ions into protoporphyrin IX [22]. Haem oxygenase (HMOX) participates in the haem degradation pathway, which converts protohaem into biliverdin, which gives eggshells a blue-green colour [6, 23, 24]. Biliverdin can be converted into bilirubin reversibly [25]. UDP-glucuronosyltransferase catalyses the formation of bilirubin β-diglucuronide [26], which is then converted into D-urobilinogen by GUSB glucuronidase beta. Finally, D-urobilinogen is oxidized into urobilin and stercobilin and then excreted out of the body. However, it remains unclear which key genes are associated with blue-green eggshell colouration in ducks.

Putian white duck and Putian black duck were both bred from Tadorna shelducks. Here, we chose a blue-green eggshell line of Putian black ducks, a white eggshell line of Putian black ducks and a white eggshell line of Putian white ducks as a research model to study genetic differences in eggshell colour formation. RNA extracted from the shell gland was analysed using RNA-seq to identify candidate genes that participate in eggshell colouration. These results will further elucidate the transcriptional mechanism of blue-green eggshell colour generation and provide a foundation for further studies of the molecular basis of eggshell colour pigmentation in avian species.

## Results

### Overview of RNA-seq results

**Fig. 1:**
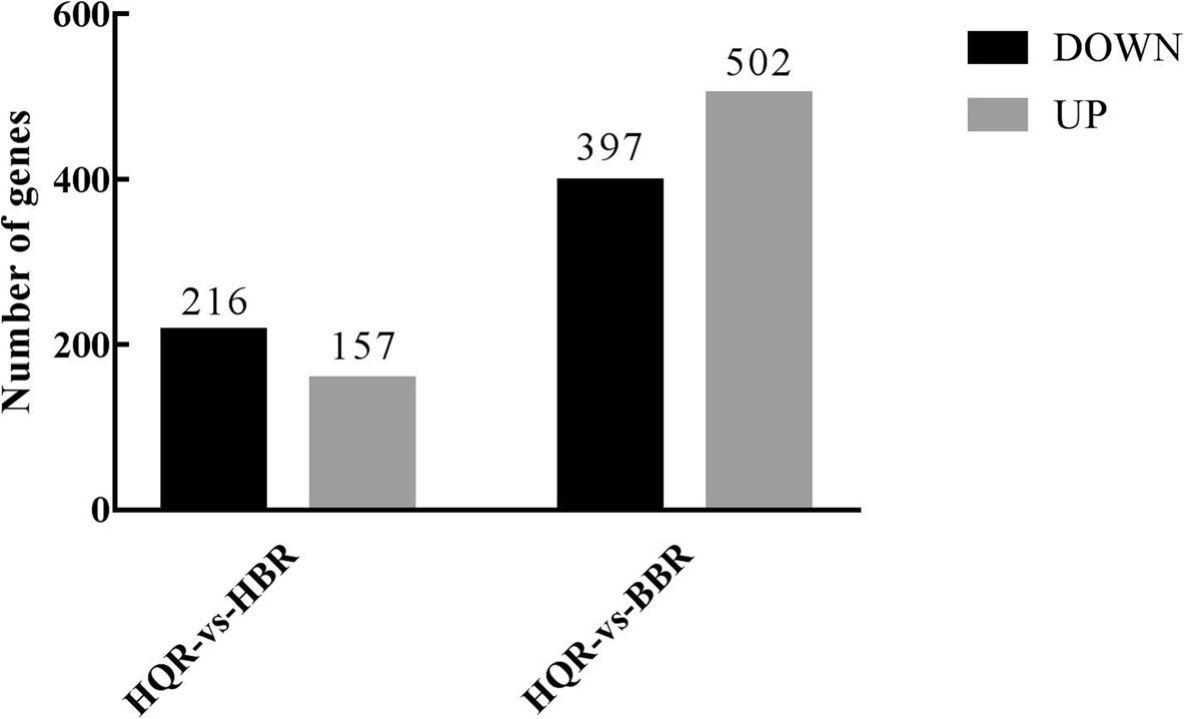
Differential gene expression statistics. The number of upregulated and downregulated genes in the HQR-vs-HBR, HQR -vs- BBR comparisons are summarized

To identify blue-green eggshell colour-related genes in Putian black ducks, we conducted a transcriptome analysis of shell glands in HQR, HBR and BBR. The main results of RNA-seq are shown in Tables 1 and 2 and Fig. 1. The number of clean reads generated from each library ranged from 64,378,314 to 84,938,126. The Q20 value was more than 97% (Table 1). After removing low-quality reads, adaptor sequences and rRNA reads, 68.35–72.47% of clean reads were mapped uniquely to the *Anas platyrhynchos* genome, and only a small proportion of them were mapped to multiple locations in the genome (Table 2). A total of 216 DEGs were downregulated, and 157 DEGs were upregulated in the HQR-vs-HBR comparison; 397 DEGs were downregulated, and 502 DEGs were upregulated in BBR-vs-HQR. Fewer DEGs were found in the HQR-vs-HBR comparison. This result may be because HQR and HBR are both Putian black duck lines, while the BBR and HQR groups are Putian white duck and Putian black duck, respectively (Fig. 1).

**Table 1:** Summary of the RNA-seq data collected from BBR, HQR and HBR

Sample name	Clean read num	Read length	Adapter (%)	Low-quality (%)	Poly-A (%)	*N* (%)	Q20%	GC%
BBR-1	70,902,122	150 + 150	52,412 (0.14%)	261,334 (0.74%)	0 (0%)	538 (0%)	97.44%	49.81%
BBR-2	68,661,202		44,700 (0.14%)	234,664 (0.68%)	0 (0%)	523 (0%)	97.50%	49.09%
BBR-3	84,938,126		58,666 (0.14%)	313,529 (0.74%)	0 (0%)	606 (0%)	97.42%	50.27%
HQR-1	79,938,462		56,120 (0.14%)	273,612 (0.68%)	0 (0%)	627 (0%)	97.51%	49.33%
HQR-2	80,656,456		53,316 (0.14%)	232,170 (0.58%)	0 (0%)	411 (0%)	98.05%	48.14%
HQR-3	83,259,050		48,330 (0.12%)	286,983 (0.69%)	0 (0%)	367 (0%)	97.87%	49.56%
HBR-1	67,865,270		43,286 (0.12%)	204,702 (0.6%)	0 (0%)	297 (0%)	98.04%	49.02%
HBR-2	64,378,314		50,400 (0.16%)	209,061 (0.65%)	0 (0%)	305 (0%)	97.94%	49.43%
HBR-3	70,599,848		41,950 (0.12%)	215,581 (0.61%)	0 (0%)	320 (0%)	98.00%	50.12%

*N*, unknown base rates higher than 10%; Q20, sequencing error rates lower than 1%

**Table 2:** Summary of clean reads and genes mapped to the reference genome from BBR, HQR and HBR

Sample name	Total reads	Mapping ratio	Multiple mapped	Unique mapped	Known genes	Known genes in three samples	Novel number	Total genes
BBR-1	69,768,600	70.61%	223,550 (0.32%)	49,043,473 (70.29%)	13,453 (89.26%)	14,318 (95.00%)	2028	15,481
BBR-2	67,525,768	71.52%	203,606 (0.30%)	48,092,609 (71.22%)	13,451 (89.24%)		1991	15,442
BBR-3	83,751,552	71.44%	224,984 (0.27%)	59,607,993 (71.17%)	13,406 (88.95%)		2013	15,419
HQR-1	78,487,372	70.30%	274,946 (0.35%)	54,903,512 (69.95%)	13,433 (89.13%)		2024	15,457
HQR-2	79,925,764	72.75%	225,898 (0.28%)	57,921,920 (72.47%)	13,393 (88.86%)		2028	15,421
HQR-3	81,339,600	69.32%	285,570 (0.35%)	56,101,799 (68.97%)	13,439 (89.17%)		2039	15,478
HBR-1	67,198,854	68.70%	234,618 (0.35%)	45,927,816 (68.35%)	13,382 (88.79%)		1978	15,360
HBR-2	63,577,054	71.01%	177,168 (0.28%)	44,968,175 (70.73%)	13,365 (88.67%)		1985	15,350
HBR-3	69,719,704	70.51%	192,410 (0.28%)	48,964,342 (70.23%)	13,315 (88.34%)		1975	15,290

### DEG functional annotation

**Fig. 2:**
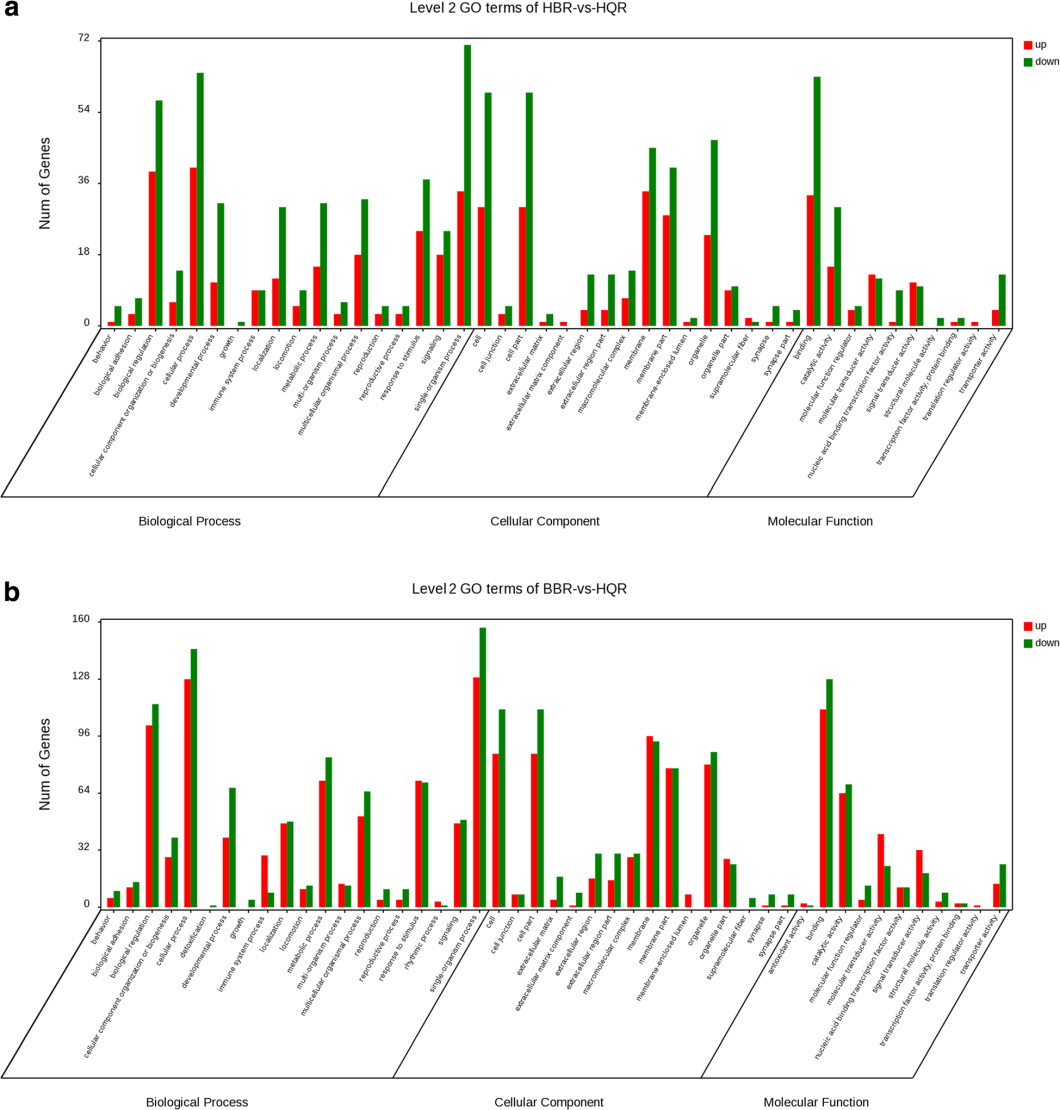
Gene Ontology (GO) classification of DEGs. **a** HQR-vs-HBR; **b** BBR-vs-HQR. The results are summarized in three main categories: biological process, cellular component and molecular function. The x-axis indicates the second-level GO term, and the y-axis indicates the number of genes

The DEGs from HQR, HBR and BBR were annotated using the Gene Ontology (GO) database, and the genes were classified into three categories. In biological process, biological regulation, cellular process and single-organism process were the most frequent terms in both comparisons; in cellular component, cell and cell part were the top terms in both comparisons; binding was observed to occur most frequently in molecular function in both comparisons (Fig. 2).

**Fig. 3:**
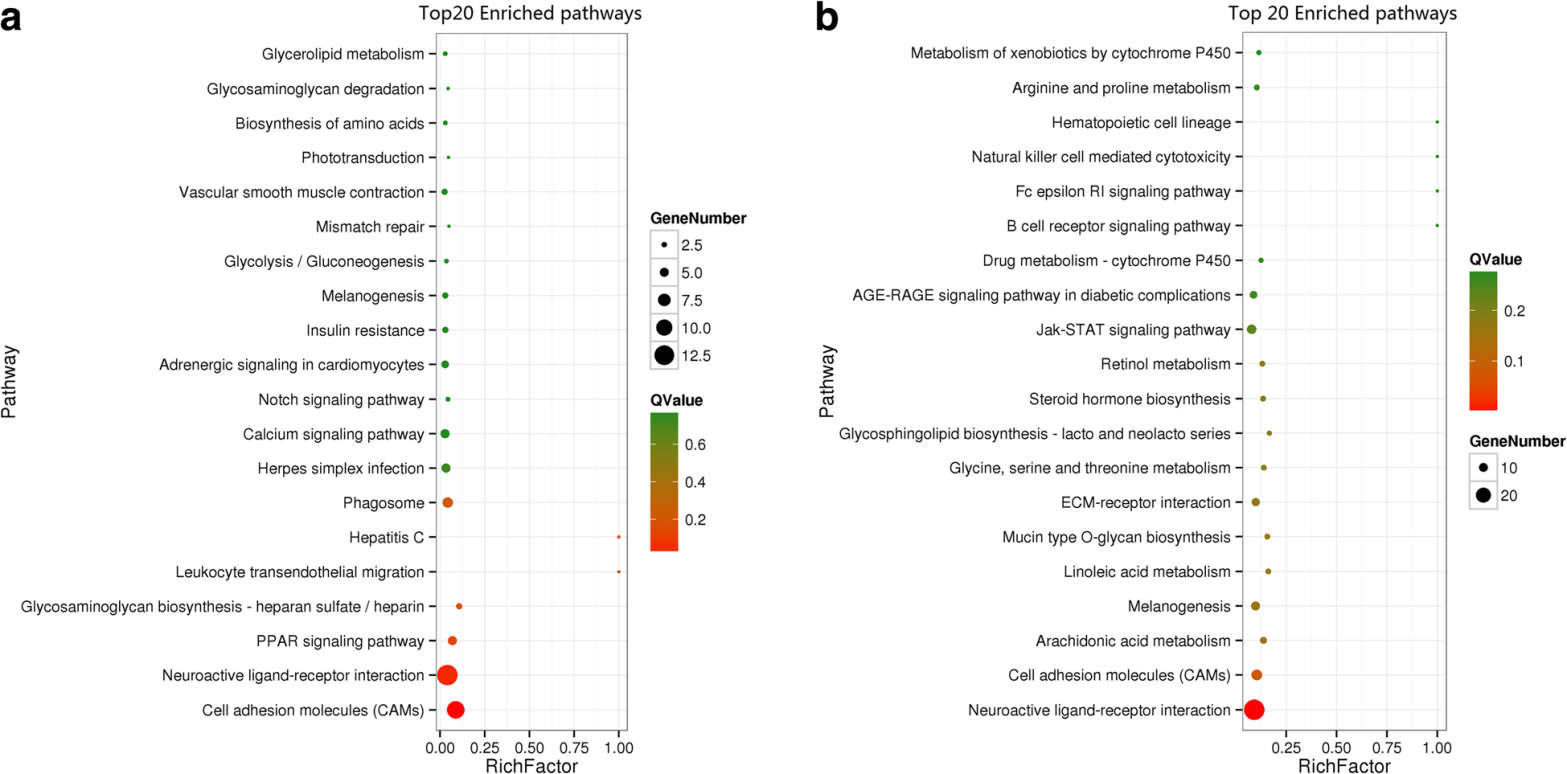
Top 20 pathways in KEGG enrichment by *Q*Value. **a** HQR-vs-HBR; **b** BBR-vs-HQR. RichFactor is the ratio of the differentially expressed number of genes in the pathway and the total number of genes in the pathway. The higher the RichFactor, the higher the degree of enrichment. *Q*Value is the *P*-value after the multiple hypothesis test correction, in the range of 0 to 1; the closer the *Q*Value is to zero, the more significant the enrichment

Pathway enrichment analysis was carried out using the Kyoto Encyclopedia of Genes and Genomes (KEGG) pathway database. As shown in Fig. 3, cell adhesion molecules (CAMs), neuroactive ligand-receptor interaction, PPAR signalling pathway, glycosaminoglycan biosynthesis-heparan sulfate/heparin, leukocyte transendothelial migration, hepatitis C and phagosome were significantly enriched in the HQR-vs-HBR comparison (*P* < 0.05). A study has found that arachidonic acid could induce oviposition and pigment secretion from the shell gland in Japanese quail [27], and glycine is the synthetic basis for protoporphyrin IX and biliverdin. In the HQR-vs-BBR comparison, arachidonic acid metabolism and glycine, serine and threonine metabolism were significantly enriched (*P* < 0.05), which suggested that they are related to eggshell pigmentation.

### Identification of blue-green eggshell colour-associated candidate genes

**Fig. 4:**
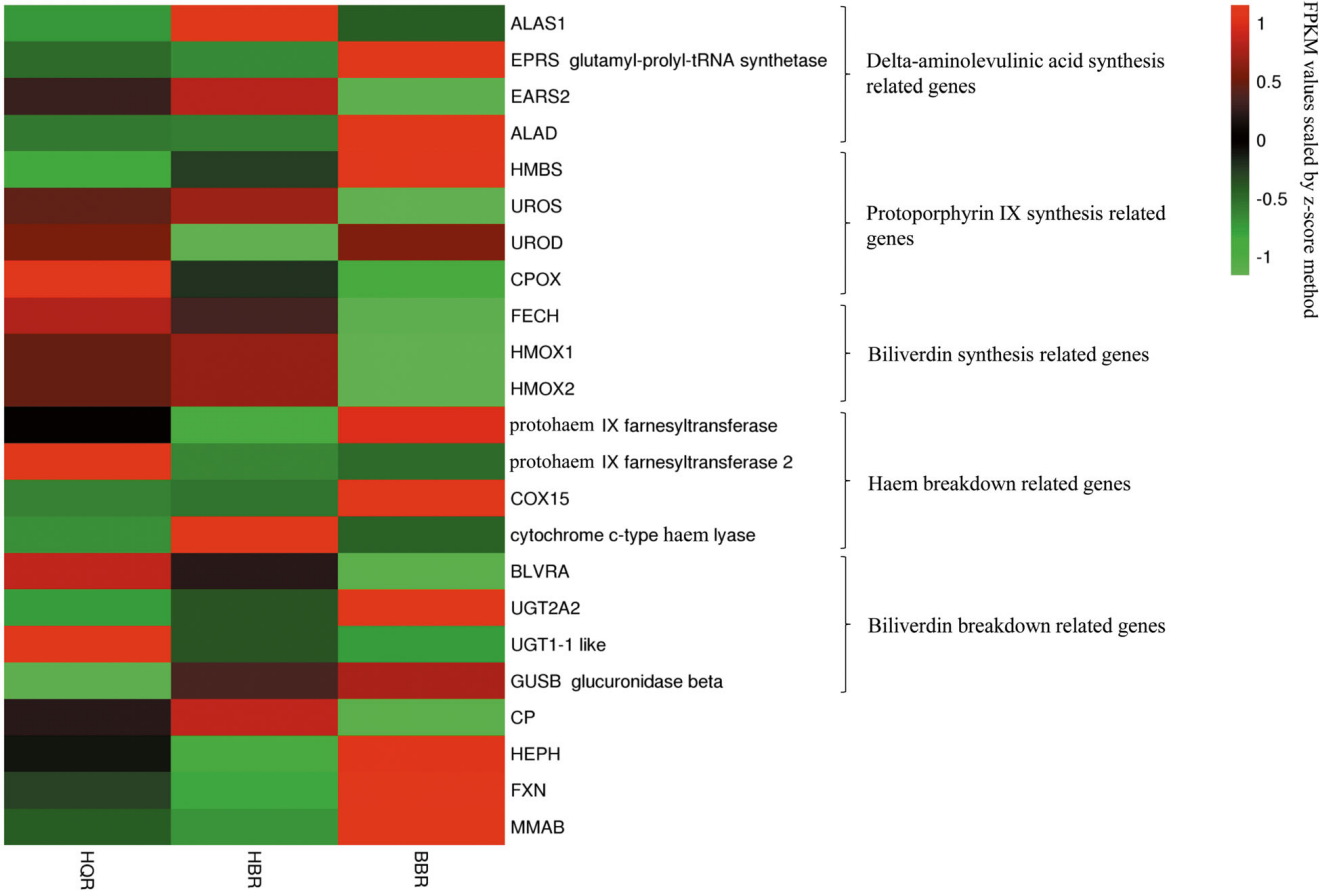
Heat map diagram of expression levels for selected eggshell colour-related genes. The Z-score scaled FPKM values for each candidate gene were used for plotting. The red represents high abundance, and the green indicates a low expression level. The columns and rows in the heat map represent samples and genes, respectively. Sample names are displayed below the heat map. The colour scale indicates fold changes in gene expression

Biliverdin is the main pigment responsible for blue-green eggshell colour. Here, a total of 4 delta-ALA synthesis-related genes, 4 protoporphyrin IX synthesis-related genes, 3 biliverdin synthesis-related genes, 4 haem breakdown-related genes, 4 biliverdin breakdown-related genes and 4 genes not directly involved in biliverdin metabolism but participating in porphyrin and chlorophyll metabolism were identified among the candidate genes in Fig. 4. ALAS1, EPRS glutamyl-prolyl-tRNA synthetase, aminolevulinate dehydratase (ALAD) and glutamyl-tRNA synthetase 2 (EARS2) participate in the synthesis of ALA [18, 28]. Meanwhile, ALAS1 is also the rate-limiting enzyme in ALA synthesis, and this gene was upregulated in the HBR group [18] and downregulated in the HQR and BBR groups. EPRS glutamyl-prolyl-tRNA synthetase and ALAD were upregulated in BBR and downregulated in HQR and HBR. EARS2 was upregulated in the HQR and HBR groups but downregulated in the BBR group. Hydroxymethylbilane synthase (HMBS), uroporphyrinogen III synthase (UROS), uroporphyrinogen decarboxylase (UROD) and coproporphyrinogen oxidase (CPOX) participate in the conversion of ALA into protoporphyrin IX [21, 29, 30]. HMBS was upregulated in BBR and downregulated in HQR and BBR. UROS was upregulated in HQR and HBR but downregulated in BBR, UROD was upregulated in HQR and BBR but downregulated in HBR, and CPOX was upregulated in HQR but downregulated in HBR and BBR. FECH, a rate-limiting enzyme that is involved in the synthesis of protohaem [31], was upregulated in the HQR and HBR groups but downregulated in the BBR group. Haem oxygenase (HMOX) is the rate-limiting enzyme that participates in the transformation of protohaem into biliverdin [24] and was upregulated in HQR and HBR but downregulated in BBR. Protohaem IX farnesyltransferase and cytochrome c oxidase assembly protein COX15 homologue (COX15) catalyse protohaem into haem A [32]. Protohaem IX farnesyltransferase and COX15 were downregulated in the HQR and HBR groups but upregulated in the BBR group. Protohaem IX farnesyltransferase 2 was upregulated in HQR and downregulated in HBR and BBR. Cytochrome c-type haem lyase participates in the transformation of protohaem into cytochrome c [33], and it was upregulated in the HBR group and downregulated in the HQR and BBR groups. The suppression of the enzymes involved in biliverdin synthesis may be the main reason that Putian white ducks lay white eggshells. In Putian black ducks, the expression levels of biliverdin synthesis-related enzymes show similar patterns in both blue-shelled and white-shelled ducks. In relation to biliverdin breakdown-related genes, BLVRA, UDP-glucuronosyltransferase (UGT) and GUSB glucuronidase beta catalyse biliverdin into D-urobilin [34, 35]. BLVRA was upregulated in the HQR and HBR groups but downregulated in the BBR group. UGT1–1-like was upregulated in HQR and downregulated in HBR and BBR, while GUSB glucuronidase beta was downregulated in HQR and upregulated in HBR and BBR. UGT2A2 was downregulated in the HQR and HBR groups but upregulated in the BBR group.

### Verification of DEGs by qPCR

**Fig. 5:**
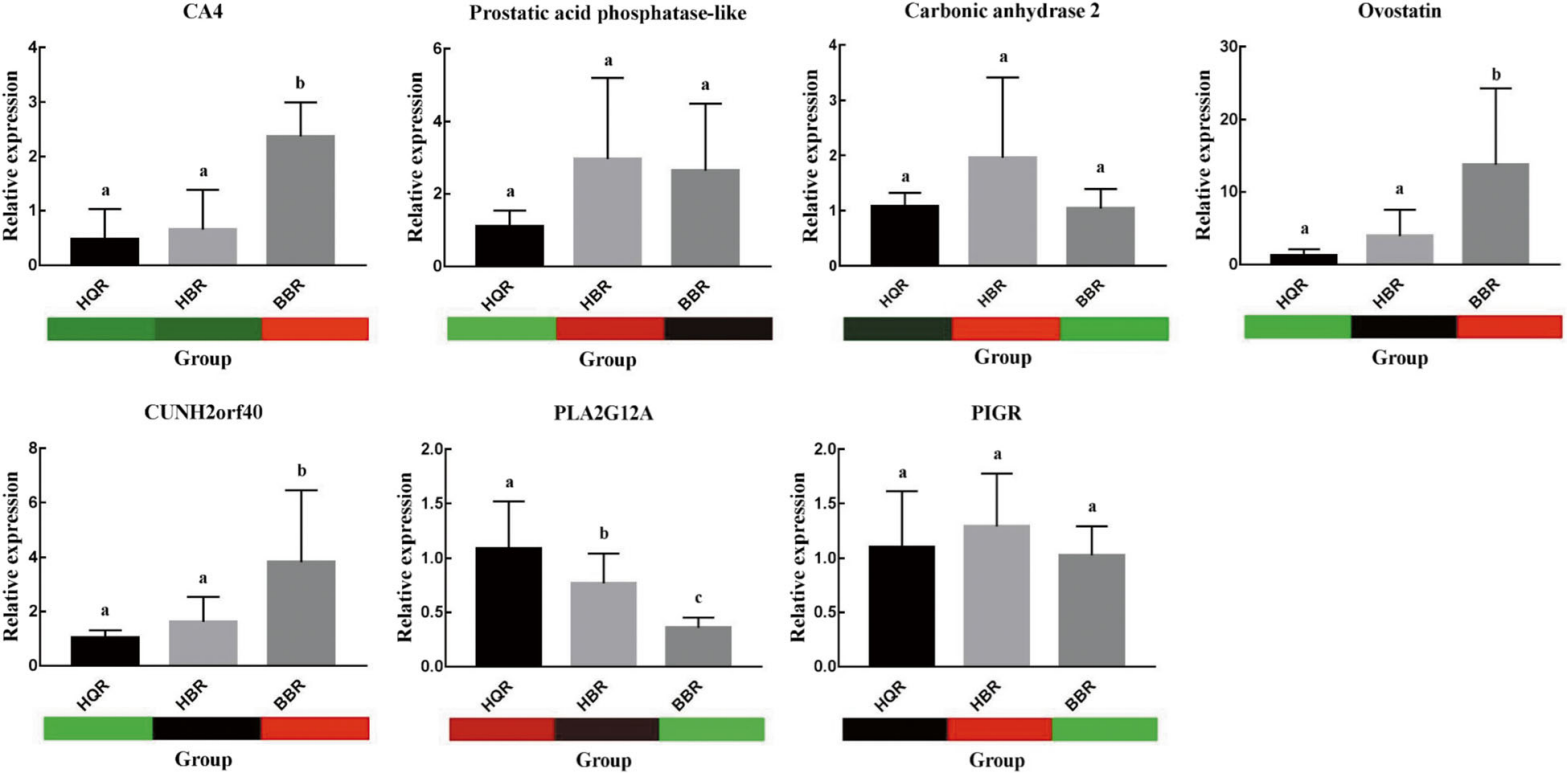
Expression levels of candidate genes by RT-qPCR and RNA-seq. The heat map shows the FPKM values for the seven selected candidate genes. Sample names are displayed below the column chart. Column height indicates the fold change in gene expression from the RT-qPCR results. RT-qPCR was performed with the primer sets in Table 3, and GAPDH was used as the reference gene. The error bars on each column indicate the SD based on three replicates. Different lowercase letters above the bars indicate statistically significant differences at *P* < 0.05 (one-way ANOVA, Duncan’s tests). Different letters indicate significant differences between groups, while the same letter represents no significant difference. The genes and gene_ids are as follows: carbonic anhydrase 4 (CA4, ncbi_101789643); prostatic acid phosphatase-like (ncbi_101805018); carbonic anhydrase 2 (ncbi_101802744); ovostatin (ncbi_101801176); CUNH2orf40 (ncbi_101802643); phospholipase A2 group XIIA (PLA2G12A, ncbi_101795972); polymeric immunoglobulin receptor-like (PIGR, ncbi_101802412)

To confirm the accuracy and reproducibility of the transcriptome analysis results, seven genes were selected for RT-qPCR validation. Primers and candidate genes are shown in Table 3. The expression profiles of seven candidate genes were determined using RT-qPCR, which were consistent with the RNA-seq results (Fig. 5), thus confirming our transcriptome analysis.

**Table 3:** PCR primers used in this study

Primer	Sequence (5′-3′)
GAPDH-F	AACGGTGACAGCCATTCCTC
GAPDH-R	CCACCACACGGTTGCTGTAT
Human C2orf40-F	CCGATGTGCAGCAGTGGTA
Human C2orf40-R	TCCTCATCATAGTGGTGCTGGT
CA4-F	AATCGGAGGTGGAGGTCTGA
CA4-R	CCACAGCATCTTCTCTTATGTGG
PIGR-F	CACTGCCGTTCATCTTCCTG
PIGR-R	TCGATCAGGCCATACACCTG
PLA2G12A-F	TGCAATCACCACGACAGATG
PLA2G12A-R	CACAAGCCTGGACACTCTCTG
Ovostatin-F	TTAGTGAGCGGCTACCAGCA
Ovostatin-R	AAGGCAGTGAGCCAGGTGTT
Carbonic anhydrase 2-F	CCACTCCACCACTGCTTGAA
Carbonic anhydrase 2-R	CGCCAGTTGTCCACCATGT
Prostatic acid phosphatase-like-F	GAGTGCTCAGGCGAGTCTTG
Prostatic acid phosphatase-like-R	TGGCACTGTGTGAACTGGAA
UDPase 2A2-F	CCTCCTACAAGGAAAATGCT
UDPase 2A2-R	CGCATGACAAATTCAATCCA
UGT1–1 like-F	ATGATCTTCTAGCTCACCCT
UGT1–1 like-R	CTGTTTTCAAGGCGTTAGAC
Protoheme IX farnesyltransferase-F	GAAAACAGGAATACGGCAAG
Protoheme IX farnesyltransferase-R	GACTTAGGTTTTGCTCGTTG
ALAS1-F	TTCCCATGGCAGATGACTAC
ALAS1-R	TATCCATCACTGCTCCACAC
BLVRA-F	AGAAAGAGGACCAGGGATGA
BLVRA-R	CGCAAAATTCGCCATTTCTC
GUSB glucuronidase-F	GCAGATATAAGCAGCATCGTC
GUSB glucuronidase-R	ATACTGCACCTTTGCTTGTG
Protoheme IX farnesyltransferase-2-F	CTTTCACGATGCTAAACGAG
Protoheme IX farnesyltransferase-2-R	CACCGTGATGGATGTAGTAG
EPRS glutamyl-prolyl-tRNA synthetase-F	GAGATGGGAAAGGTCATTGT
EPRS glutamyl-prolyl-tRNA synthetase-R	ATTCGTGTCATCAAACCTCA
EARS2-F	TATGCTTACTTGTGGGTCAG
EARS2-R	GCTGCTGTACTTAGTCTCTC
UROD-F	TTTTCTCTGACATCTTGGTGG
UROD-R	CATGTAGGACATCAGCGTC
CP-F	GGACCCATTATCAAGGCAGA
CP-R	GTTCTTGCGGTAACTCACAC
FECH-F	CTCACAAGTACTACATCGGG
FECH-R	TACTCCACTTCATCTTTGGC
COX15-F	CTGGGCTGGTACATGGTCAA
COX15-R	GGTCTCGGGTAACTGGTGTC
ALAD-F	AAACCTTTACCGGGATTTCT
ALAD-R	TTCCTCAAAGATCCTCCTGT
HMBS-F	ATTTGCCTGTTGCGTTTAGG
HMBS-R	CCATTCTCCTTCAGCGTTTC
FXN-F	TTTCTTTGAGGACCTAACCG
FXN-R	AGAATACACCCAATTCCGTC
Cytochrome c-type heme lyase-F	GGTTTCTGGTTCTTCTGACT
Cytochrome c-type heme lyase-R	GCGCTCATGTAAAAACCATT
UROS-F	TATGGGGGCCTAGTTTTCAC
UROS-R	GTCTTTTCTCCTTGTGGGGA
HEPH-F	GCAGACATGATCCCCAGTAA
HEPH-R	GAAGGTCCATAGTCCCACTG
CPOX-F	CACTAAACAGTGGTGGTTTG
CPOX-R	AAGAATATGCCTCCAATCCC
MMAB-F	CTGGAGCAGTGGATTGACA
MMAB-R	TTCCCCTGCTTGTACTAACG
HMOX1-F	ATACGTTGAAAGGCTCCACT
HMOX1-R	TGGGCGATTTTCTTCAACAC
HMOX2-F	AGTCTACACATGCTTGGCTT
HMOX2-R	AAATTGAGTGCTCTTCGCTG

### Transcript levels of porphyrin and chlorophyll metabolism-related genes by qPCR

**Fig. 6:**
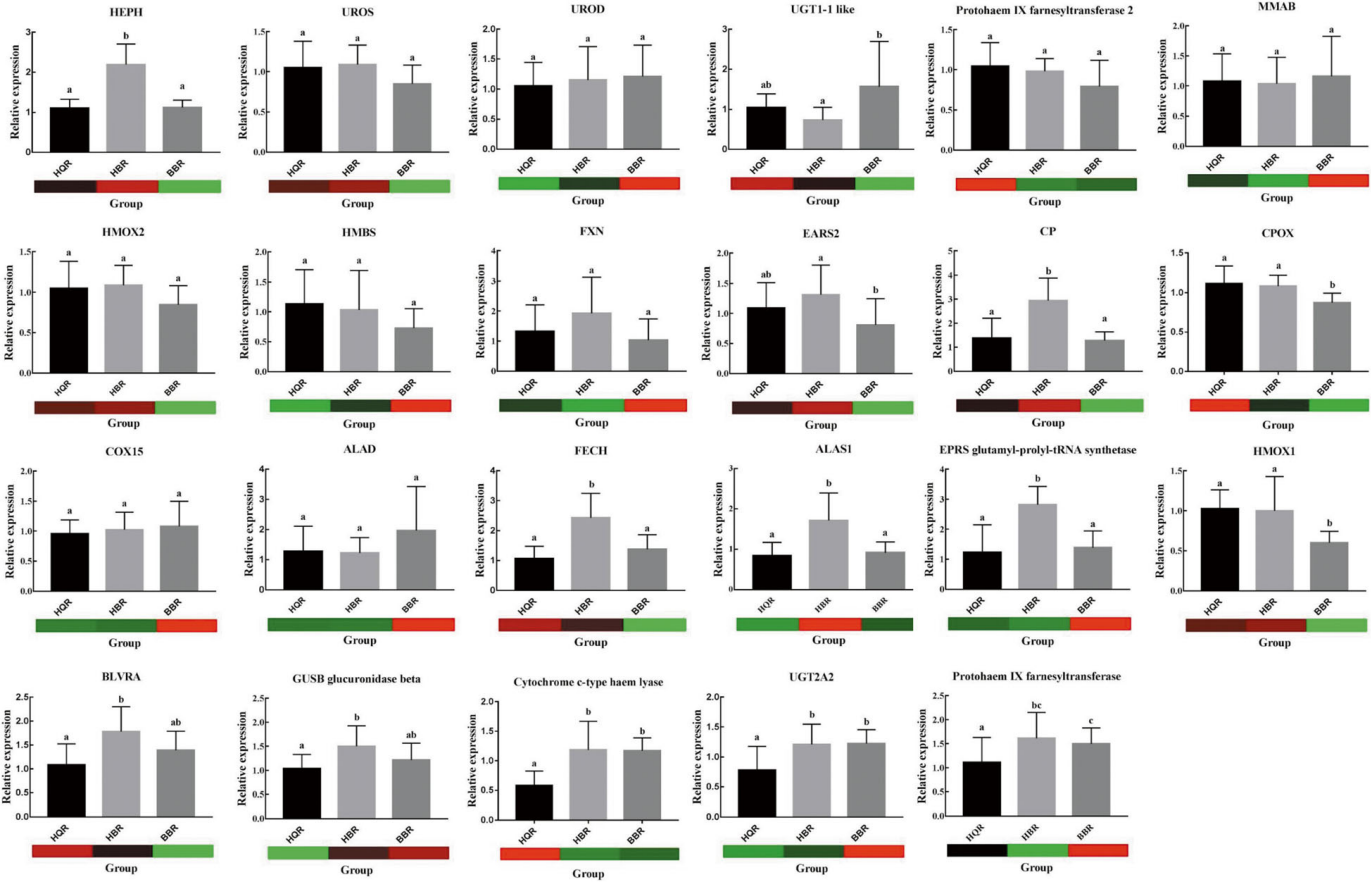
Expression levels of porphyrin and chlorophyll metabolism-related genes by RT-qPCR and RNA-seq. The heat map shows the FPKM values for the 23 selected candidate genes. Sample names are displayed below the column charts. Column height indicates the fold change in gene expression from the RT-qPCR results. RT-qPCR was performed with the primer sets in Table 2, and GAPDH was used as the reference gene. The error bars on each column indicate the SD based on three replicates. Different lowercase letters above the bars indicate statistically significant differences at *P* < 0.05 (one-way ANOVA, Duncan’s tests). Different letters indicate significant differences between groups, while the same letter represents no significant difference. The genes and the gene_ids are as follows: HEPH (ncbi_101803569); UROS (ncbi_101803009); UROD (ncbi_101794950); UGT1–1-like (ncbi_101802378); protohaem IX farnesyltransferase 2 (ncbi_101790381); MMAB (ncbi_101804472); HMOX2 (ncbi_101801310); HMBS (ncbi_101799203); FXN (ncbi_101800676); EARS2 (ncbi_101794546); CP (ncbi_101795788); CPOX (ncbi_101804003); COX15 (ncbi_101800616); ALAD (ncbi_101797189); FECH (ncbi_101796583); ALAS1 (ncbi_101793120); EPRS glutamyl-prolyl-tRNA synthetase (ncbi_101794263); HMOX1 (ncbi_101805057); BLVRA (ncbi_101793149); GUSB glucuronidase beta (ncbi_101793510); Cytochrome c-type haem lyase (ncbi_101802937); UGT2A2 (ncbi_101796668); Protohaem IX farnesyltransferase (ncbi_101790381)

In the HQR-vs-BBR comparison, UGT2A2 and UGT1–1-like, which participate in biliverdin breakdown, were two DEGs. Here, 23 porphyrin and chlorophyll metabolism-related genes were analysed by RT-qPCR (Fig. 6). None of these genes were differentially expressed in the RNA-seq results except for UGT2A2 and UGT1–1-like, while some showed significant differences in the RT-qPCR results. Compared with the RT-qPCR results, the expression profiles of 10 genes were not consistent with the RNA-seq data. In the RT-qPCR results, ALAS1 and EPRS glutamyl-prolyl-tRNA synthetase were significantly upregulated in the HBR group compared with the HQR and BBR groups (*P* < 0.05). HMOX1 was significantly downregulated in BBR compared with HQR and HBR (*P* < 0.05). BLVRA, GUSB, cytochrome c-type haem lyase, protohaem IX farnesyltransferase and UGT2A2 were significantly upregulated in the HBR and BBR groups compared with the HQR group (*P* < 0.05). In HBR, 5 biliverdin breakdown-related genes were upregulated. In BBR, 1 biliverdin synthesis-related gene was downregulated, while 5 biliverdin breakdown-related genes were downregulated. The expression patterns of these genes may be the main cause of white eggshell colour.

## Discussion

To help elucidate the molecular basis of eggshell pigmentation, we obtained a global view of transcriptomes from the shell glands of the HQR, HBR and HQR groups. However, none of these DEGs were directly involved in the biosynthesis or transportation of biliverdin in the HBR and HQR groups, while UGT2A2 and UGT1–1-like, which participate in biliverdin breakdown, were two DEGs in the HQR-vs-BBR comparison. In HQR-vs-HBR, CAMs, neuroactive ligand-receptor interaction, the PPAR signalling pathway, glycosaminoglycan biosynthesis-heparan sulfate/heparin, leukocyte transendothelial migration, hepatitis C and phagosome were significantly enriched. None of these pathways or DEGs were related to the synthesis of biliverdin. In the HQR-vs-BBR comparison, arachidonic acid metabolism and glycine, serine and threonine metabolism were significantly enriched (*P* < 0.05). Arachidonic acid participates in the local synthesis of prostaglandin. A previous study has found that both prostaglandin and arachidonic acid can induce pigment secretion, which is consistent with our results [27].

**Fig. 7:**
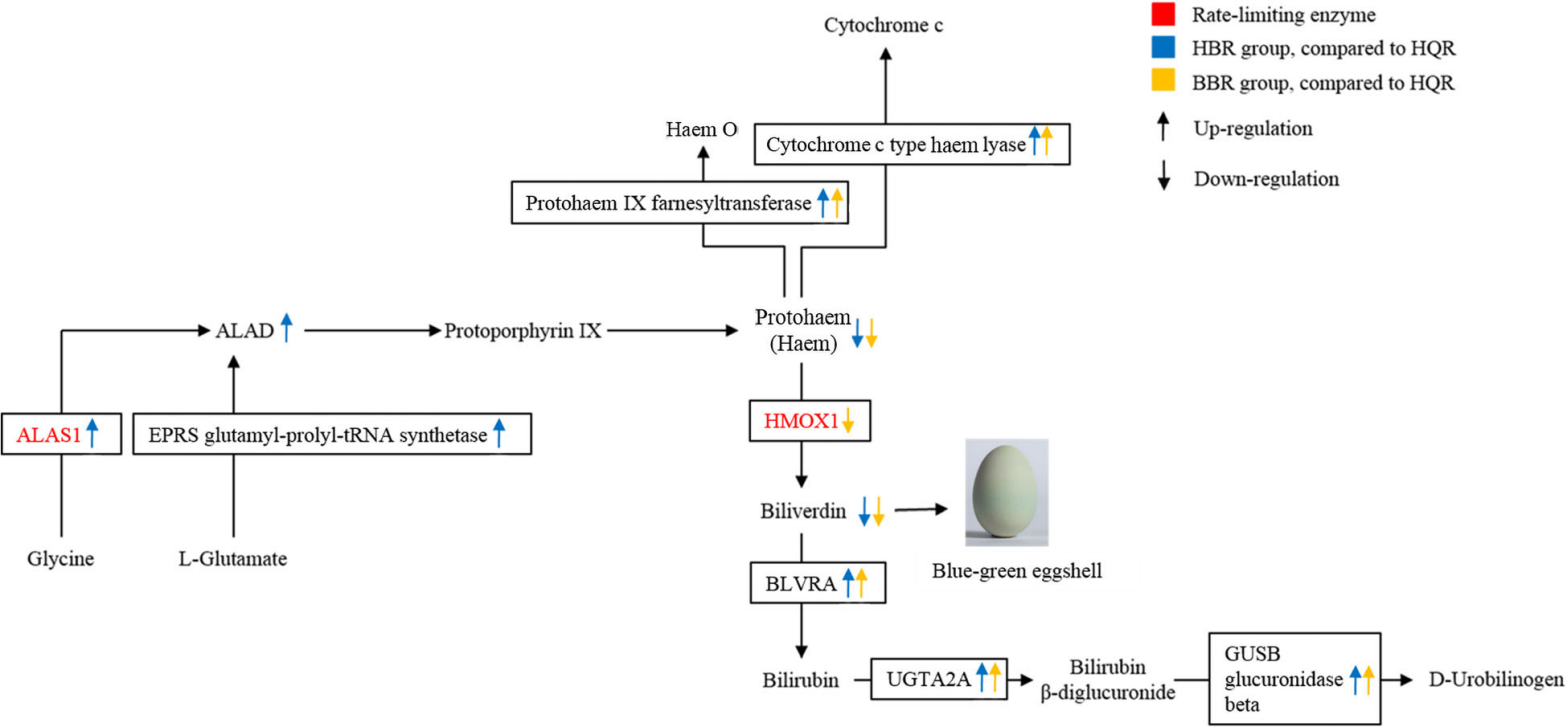
A hypothesis explaining that differential expression of biliverdin synthesis-related genes can cause blue-green eggshell colour. Gene names shown in red represent rate-limiting enzymes. The blue upward arrows represent upregulation in HBR compared to HQR. The blue downward arrow represents downregulation in HBR compared to HQR. The yellow upward arrows represent upregulation in BBR compared to HQR. The yellow downward arrow represents downregulation in HBR compared to HQR

Next, we analysed the expression level of biliverdin-related genes by RT-qPCR. ALAS1 and EPRS glutamyl-prolyl-tRNA synthetase participate in the biosynthesis of ALA, which is utilized for the biosynthesis of protoporphyrin IX and biliverdin. It is well established that HMOX is the rate-limiting enzyme in the catabolism of haem into biliverdin, free iron, and carbon monoxide [36]. A study has also found that Dongxiang blue-shelled chickens have higher HMOX1 expression levels and enzymatic activity than do Dongxiang brown-shelled chickens [37]. HMOX1 was significantly downregulated in BBR compared to HQR and HBR, which is consistent with the findings. However, HMOX1 and HMOX2 were not significantly different between the HQR and HBR groups. This result is consistent with the finding of Wang et al. [37] that HMOX1 and HMOX2 were not significantly different between the blue-green-shelled and white-shelled duck groups. BLVRA, GUSB, cytochrome c-type haem lyase, protohaem IX farnesyltransferase and UGT2A2 were significantly upregulated in HBR and BBR compared with HQR. Thus, we speculate that HQR and HBR can produce biliverdin, but HBR cannot accumulate it. This result is also similar to the findings of Japanese scientists, who found that both red coral and blue coral can produce biliverdin, but red coral cannot accumulate biliverdin in its skeleton [38]. Compared with the HQR group, the BBR group produced less biliverdin and did not accumulate it (Fig. 7).

## Conclusions

This study conducted a comparative transcriptome analysis of the shell glands of Putian white ducks and Putian black ducks. In the HQR-vs-HBR comparison, none of the DEGs were directly involved in the eggshell pigmentation process. In the HQR-vs-BBR comparison, UGT2A2 and UGT1–1-like were considered to be two DEGs related to biliverdin breakdown. In the KEGG analysis, glycine, serine and threonine metabolism and arachidonic acid metabolism may play potential roles in the synthesis and secretion of pigments, based on the HQR-vs-BBR comparison.

From the RT-qPCR results, 5 biliverdin breakdown-related genes were upregulated in the HBR group. In the BBR group, 1 biliverdin synthesis-related gene was downregulated, and 5 biliverdin breakdown-related genes were downregulated. Thus, we can speculate that HQR and HBR can produce biliverdin, but HBR cannot accumulate it. Compared with HQR, BBR produces less biliverdin and cannot accumulate it. These results will provide new insight into further functional genomic research on the mechanism of blue-green eggshell colouration.

## Methods

### Ethics statement

All animal experiments were reviewed and approved by the Institutional Animal Care and Use Committee of the College of Animal Science, Fujian Agriculture and Forestry University. All of the following procedures were performed strictly according to the regulations and guidelines established by the committee.

### Animals and tissue collection

In this study, the Putian black ducks and Putian white ducks were all bred by Shishi Conservation and Research Centre of Waterfowl Genetic Resources. We randomly collected nine Putian white ducks that produced white eggshells, nine Putian black ducks that produced white eggshells and nine Putian black ducks that produced blue-green eggshells. Three shell gland samples of the same phenotype were mixed in equal amounts for further mRNA sequencing. According to their individual oviposition routines, ducks were stunned and killed 3–5 h before estimated oviposition. Then, the shell gland samples were washed with 0.9% isotonic saline and preserved in liquid nitrogen for further RNA extraction. All the ducks were slaughtered at the same time, and their intact shell glands were harvested.

### RNA extraction, library construction and transcriptome sequencing

Total RNA from 27 shell gland samples was isolated using TRIzol reagent (Invitrogen, CA, USA) following the manufacturer’s protocol. RNA concentration and purity were checked with a Nanodrop 2000c (Thermo, CA, USA). Poly-A-containing mRNA molecules were purified from total RNA using oligo-dT-attached magnetic beads and fragmented using fragmentation buffer. First-strand cDNA synthesis was performed using a random hexamer primer and reverse transcriptase followed by second-strand cDNA synthesis using DNA polymerase and RNase H. The product was purified using a QIAquick PCR purification kit (Qiagen, Hilden, Germany). The cDNA fragments then underwent an end repair process, the addition of a single ‘A’ base, and adapter ligation. The products were then purified and enriched with PCR to create the final cDNA library. Sequencing was performed using Illumina® HiSeq 2500 at Gene Denovo Bioinformatics Technologies Co. Ltd., Guangzhou, China.

### Sequence read quality control, mapping, and annotation

Quality of the raw reads was assessed using FastQC [39]. Raw reads that contained adapter, more than 10% unknown bases or low-quality reads were removed to obtain high-quality clean reads. Next, rRNA was removed from the high-quality clean reads using Bowtie by mapping to a ribosome database [40]. The remaining high-quality clean reads were aligned to the reference genome (*Anas platyrhynchos*) using TopHat2 [41]. Subsequently, the Cufflinks reference annotation based transcript (RABT) assembly method was used to assemble these mapped reads into possible transcripts and then generate a final transcriptome assembly [42, 43]. The GO and KEGG enrichment analyses were performed with a *Q*-value cut-off of 0.05.

### RNA-seq analysis

In this study, the read numbers mapped to each gene were counted using edgeR. The FPKM (fragments per kilobase of transcript per million mapped reads) value of each gene was calculated based on the length of the gene and the read counts mapped to this gene. The resulting *P*-values were adjusted using the Benjamini and Hochberg approach for controlling the false discovery rate (FDR) [44]. Genes with an adjusted *P*-value of < 0.05 and | Log2 (fold change) | > 1 were identified as differentially expressed genes (DEGs) by edgeR.

### Real-time PCR assay

cDNA synthesis was performed using the GoScript™ Reverse Transcription System (Promega, USA) following the manufacturer’s instructions. Quantitative gene expression was assessed by real-time PCR with a CFX96 Touch Deep Well Real-Time PCR Detection System (Bio-Rad, USA). Target genes were amplified using the primers in Table 3: GAPDH was employed as an endogenous control. The reaction mixture contained 6.25 μL 2× GoTaq® qPCR Master Mix (Promega, USA) following the manufacturer’s instructions. Each reaction was run in triplicate. The qRT-PCR conditions were an initial predenaturation step at 95 °C for 3 min, followed by 39 cycles of 95 °C for 20 s and 60 °C for 30 s. Fluorescence data were collected and analysed with the Bio-Rad CFX Manager software and normalized to those of GAPDH. Melting curves were generated after the qPCR was completed.

### Real-time PCR analysis

Differences between groups were analysed using ANOVA. Experimental data are expressed as the mean ± SE from three independent experiments. *P*-values less than 0.05 were considered statistically significant. Statistical analyses were performed by using SPSS version 10.0.

## Abbreviations

ALA: Delta-aminolevulinic acid
ALAD: Aminolevulinate dehydratase
ALAS1: Delta-aminolevulinic acid synthase 1
BBR: White-shelled ducks from Putian white duck population
BLVRA: Biliverdin reductase A
COX15: Cytochrome c oxidase assembly protein COX15 homologue
CPOX: Coproporphyrinogen oxidase
DEGs: Differentially expressed genes
EARS2: Glutamyl-tRNA synthetase 2
FECH: Ferrochelatase
HBR: White-shelled ducks from Putian black duck population
HMBS: Hydroxymethylbilane synthase
HMOX: Haem oxygenase
HQR: Blue-green-shelled ducks from Putian black duck population
UGT: UDP-glucuronosyltransferase
UGT1–1-like: UDP-glucuronosyltransferase 1–1-like
UGT2A2: UDP-glucuronosyltransferase 2A2
UROD: Uroporphyrinogen decarboxylase
UROS: Uroporphyrinogen III synthase

## References

[1] Kilner RM (2006). The evolution of egg colour and patterning in birds. Biol Rev Camb Philos Soc.

[2] Samiullah S, Roberts J, Chousalkar K (2016). Oviposition time, flock age, and egg position in clutch in relation to brown eggshell color in laying hens. Poult Sci.

[3] Bakken GS, Vanderbilt VC, Buttemer WA, Dawson WR (1978). Avian eggs: thermoregulatory value of very high near-infrared reflectance. Science.

[4] Ishikawa S, Suzuki K, Fukuda E, Arihara K, Yamamoto Y, Mukai T, Itoh M (2010). Photodynamic antimicrobial activity of avian eggshell pigments. FEBS Lett.

[5] Samiullah S, Roberts JR, Chousalkar K (2015). Eggshell color in brown-egg laying hens - a review. Poult Sci.

[6] Hargitai R, Boross N, Hamori S, Neuberger E, Nyiri Z (2017). Eggshell Biliverdin and Protoporphyrin pigments in a songbird: are they derived from erythrocytes, blood plasma, or the Shell gland?. Physiol Biochem Zool.

[7] Afonso S, Vanore G, Batlle A (1999). Protoporphyrin IX and oxidative stress. Free Radic Res.

[8] Stocker R, Yamamoto Y, McDonagh AF, Glazer AN, Ames BN (1987). Bilirubin is an antioxidant of possible physiological importance. Science.

[9] Soler JJ, Moreno J, Aviles JM, Moller AP (2005). Blue and green egg-color intensity is associated with parental effort and mating system in passerines: support for the sexual selection hypothesis. Evolution.

[10] Stoddard MC, Fayet AL, Kilner RM, Hinde CA (2012). Egg speckling patterns do not advertise offspring quality or influence male provisioning in great tits. PLoS One.

[11] Duval C, Cassey P, Miksik I, Reynolds SJ, Spencer KA (2013). Condition-dependent strategies of eggshell pigmentation: an experimental study of Japanese quail (Coturnix coturnix japonica). J Exp Biol.

[12] Yuan QY, Lu LZ (2007). Progresses in inheritance of genes for avian eggshell color. Yi Chuan.

[13] Gorchein A, Lord G, Lim CK (2012). Isolation and characterization of free haem from the shell gland of quail and hen. Biomed Chromatogr.

[14] Li G, Chen S, Duan Z, Qu L, Xu G, Yang N (2013). Comparison of protoporphyrin IX content and related gene expression in the tissues of chickens laying brown-shelled eggs. Poult Sci.

[15] Zhao R, Xu GY, Liu ZZ, Li JY, Yang N (2006). A study on eggshell pigmentation: biliverdin in blue-shelled chickens. Poult Sci.

[16] Kennedy GY, Vevers HG (1973). Eggshell pigments of the Araucano fowl. Comp Biochem Physiol B.

[17] Guernsey DL, Jiang H, Campagna DR, Evans SC, Ferguson M, Kellogg MD, Lachance M, Matsuoka M, Nightingale M, Rideout A (2009). Mutations in mitochondrial carrier family gene SLC25A38 cause nonsyndromic autosomal recessive congenital sideroblastic anemia. Nat Genet.

[18] Laver WG, Neuberger A, Udenfriend S (1958). Initial stages in the biosynthesis of porphyrins. I. The formation of delta-am-inolaevulic acid by particles obtained from chicken erythrocytes. Biochem J.

[19] Samiullah S, Roberts J, Wu SB (2017). Downregulation of ALAS1 by nicarbazin treatment underlies the reduced synthesis of protoporphyrin IX in shell gland of laying hens. Sci Rep.

[20] Quigley JG, Yang Z, Worthington MT, Phillips JD, Sabo KM, Sabath DE, Berg CL, Sassa S, Wood BL, Abkowitz JL (2004). Identification of a human heme exporter that is essential for erythropoiesis. Cell.

[21] Takahashi S, Taketani S, Akasaka JE, Kobayashi A, Hayashi N, Yamamoto M, Nagai T (1998). Differential regulation of coproporphyrinogen oxidase gene between erythroid and nonerythroid cells. Blood.

[22] Al-Karadaghi S, Hansson M, Nikonov S, Jonsson B, Hederstedt L (1997). Crystal structure of ferrochelatase: the terminal enzyme in heme biosynthesis. Structure.

[23] Noguchi M, Yoshida T, Kikuchi G (1979). Specific requirement of NADPH-cytochrome c reductase for the microsomal heme oxygenase reaction yielding biliverdin IX alpha. FEBS Lett.

[24] Spencer AL, Bagai I, Becker DF, Zuiderweg ER, Ragsdale SW (2014). Protein/protein interactions in the mammalian heme degradation pathway: heme oxygenase-2, cytochrome P450 reductase, and biliverdin reductase. J Biol Chem.

[25] Schluchter WM, Glazer AN (1997). Characterization of cyanobacterial biliverdin reductase. Conversion of biliverdin to bilirubin is important for normal phycobiliprotein biosynthesis. J Biol Chem.

[26] Peters WH, Jansen PL (1986). Microsomal UDP-glucuronyltransferase-catalyzed bilirubin diglucuronide formation in human liver. J Hepatol.

[27] Soh T, Koga O (1999). Effects of phosphate, prostaglandins, arachidonic acid and arginine vasotocin on oviposition and pigment secretion from the shell gland in Japanese quail. Br Poult Sci.

[28] Park SG, Schimmel P, Kim S (2008). Aminoacyl tRNA synthetases and their connections to disease. Proc Natl Acad Sci U S A.

[29] Johansson P, Hederstedt L (1999). Organization of genes for tetrapyrrole biosynthesis in gram--positive bacteria. Microbiology.

[30] Tsai SF, Bishop DF, Desnick RJ (1988). Human uroporphyrinogen III synthase: molecular cloning, nucleotide sequence, and expression of a full-length cDNA. Proc Natl Acad Sci U S A.

[31] Rutherford T, Thompson GG, Moore MR (1979). Heme biosynthesis in friend erythroleukemia cells: control by ferrochelatase. Proc Natl Acad Sci U S A.

[32] Swenson S, Cannon A, Harris NJ, Taylor NG, Fox JL, Khalimonchuk O (2016). Analysis of oligomerization properties of Heme a synthase provides insights into its function in eukaryotes. J Biol Chem.

[33] Lill R, Stuart RA, Drygas ME, Nargang FE, Neupert W (1992). Import of cytochrome c heme lyase into mitochondria: a novel pathway into the intermembrane space. EMBO J.

[34] Komuro A, Tobe T, Nakano Y, Yamaguchi T, Tomita M (1996). Cloning and characterization of the cDNA encoding human biliverdin-IX alpha reductase. Biochim Biophys Acta.

[35] Green MD, Falany CN, Kirkpatrick RB, Tephly TR (1985). Strain differences in purified rat hepatic 3 alpha-hydroxysteroid UDP-glucuronosyltransferase. Biochem J.

[36] Maines MD (1997). The heme oxygenase system: a regulator of second messenger gases. Annu Rev Pharmacol Toxicol.

[37] Wang ZP, Liu RF, Wang AR, Li JY, Deng XM (2011). Expression and activity analysis reveal that heme oxygenase (decycling) 1 is associated with blue egg formation. Poult Sci.

[38] Hongo Y, Yasuda N, Naga IS (2017). Identification of genes for synthesis of the blue pigment, Biliverdin IXalpha, in the blue coral Heliopora coerulea. Biol Bull.

[39] Cock PJ, Fields CJ, Goto N, Heuer ML, Rice PM (2010). The sanger FASTQ file format for sequences with quality scores, and the Solexa/Illumina FASTQ variants. Nucleic Acids Res.

[40] Langmead B, Trapnell C, Pop M, Salzberg SL (2009). Ultrafast and memory-efficient alignment of short DNA sequences to the human genome. Genome Biol.

[41] Kim D, Pertea G, Trapnell C, Pimentel H, Kelley R, Salzberg SL (2013). TopHat2: accurate alignment of transcriptomes in the presence of insertions, deletions and gene fusions. Genome Biol.

[42] Trapnell C, Williams BA, Pertea G, Mortazavi A, Kwan G, van Baren MJ, Salzberg SL, Wold BJ, Pachter L (2010). Transcript assembly and quantification by RNA-Seq reveals unannotated transcripts and isoform switching during cell differentiation. Nat Biotechnol.

[43] Roberts A, Pimentel H, Trapnell C, Pachter L (2011). Identification of novel transcripts in annotated genomes using RNA-Seq. Bioinformatics.

[44] Lesack K, Naugler C (2011). An open-source software program for performing Bonferroni and related corrections for multiple comparisons. J Pathol Inform.

